# Outcomes of singleton preterm very low birth weight infants born to mothers with pregnancy-induced hypertension

**DOI:** 10.1038/s41598-023-33206-y

**Published:** 2023-04-13

**Authors:** Hye-Rim Kim, Byoung Kook Lee

**Affiliations:** 1 Department of Pediatrics, Bundang CHA Medical Center, CHA University, Seongnam, Korea; 2 Department of Pediatrics, Chungnam National University Sejong Hospital, 20, Bodeum 7-ro, Sejong-si, Sejong, Republic of Korea

**Keywords:** Neonatology, Respiratory distress syndrome, Preterm birth

## Abstract

The association between maternal pregnancy-induced hypertension (PIH) and neonatal mortality and morbidities in preterm infants has not been consistent. This study aimed to evaluate the influence of maternal PIH on mortality and morbidities in singleton infants with very low birth weight born before 30 weeks of gestational age using the Korean Neonatal Network (KNN) database. A total of 5340 singleton infants with very low birth weight were registered in the KNN registry, who were born at 23^+0^ to 29^+6^ weeks of gestational age between January 2015 and December 2020. Baseline characteristics and neonatal mortality and morbidities were compared between infants with PIH and non-PIH mothers. After adjustment for potential confounders, infants with PIH mothers had significantly higher odds of respiratory distress syndrome (OR 1.983; 95% CI 1.285–3.061, *p* = 0.002) and bronchopulmonary dysplasia (OR 1.458; 95% CI 1.190–1.785, *p* < 0.001), and severe bronchopulmonary dysplasia (OR 1.411; 95% CI 1.163–1.713, *p* < 0.001) than infants with non-PIH mothers, while there were no significant differences in severe intraventricular hemorrhage, periventricular leukomalacia, retinopathy of prematurity, or death during neonatal intensive care unit admission between infants with PIH and non-PIH mothers. This study showed that preterm infants with PIH mothers had an increased risk of neonatal respiratory morbidities, including respiratory distress syndrome and bronchopulmonary dysplasia.

## Introduction

Pregnancy-induced hypertension (PIH) affects approximately 5–10% of pregnancies^1^. PIH is associated with severe complications such as placental abruption, HELLP (hemolysis, elevated liver enzymes, and low platelet count) syndrome, preterm birth, intrauterine growth retardation, and even fetal or maternal death^2–5^. Of preterm births in particular, up to 20% are due to hypertensive disorders of pregnancy^6^. Moreover, recent reports have indicated rising rates of PIH as a result of changing maternal characteristics^7^. Treatment of PIH depends on blood pressure levels, gestational age (GA), presence of symptoms and associated risk factors^5^. Given the high risk of neonatal mortality and morbidity in preterm infants, finding the proper balance between delivery in the second trimester and continuation of pregnancy in mothers with PIH is crucial.

The association between maternal PIH and neonatal mortality and morbidities in preterm infants has been examined^8–17^. However, the findings regarding neonatal mortality and morbidities in preterm infants born to PIH mothers have not been consistent. Some studies have demonstrated that preterm infants born to mothers with PIH have an increased risk of several morbidities, including respiratory distress syndrome (RDS) and bronchopulmonary dysplasia (BPD)^8–11^. Others have demonstrated a decreased risk of mortality, intraventricular hemorrhage (IVH) and periventricular leukomalacia (PVL) in preterm infants with PIH mothers^8,9,12–17^.

The purpose of this study was to evaluate the influence of maternal PIH on mortality and morbidities in singleton infants with very low birth weight (VLBW) born before 30 weeks GA using the Korean Neonatal Network (KNN) database.

## Methods

### Study population

This study was based on an analysis of prospectively collected data from 70 neonatal intensive care units (NICUs) participating in the KNN. The KNN registry includes approximately 70% of all infants with VLBW (< 1500 g) born in South Korea^18^. The singleton infants with VLBW registered in the KNN registry who were born at 23^+0^ to 29^+6^ weeks GA from January 2015 to December 2020 were enrolled. The infants with major congenital anomalies or infants born to mothers with chronic hypertension were excluded. Neonatal mortality, and morbidities between infants with PIH mothers or non-PIH mothers were analyzed.

### Data collection

The KNN is a nationwide multicenter registry of infants with VLBW that prospectively collects demographic and clinical data using a standardized operating procedure^18^. The maternal data included age, PIH, diabetes, premature rupture of membrane (PROM), the use of antenatal corticosteroids, and delivery mode. The neonatal data included GA, birth weight, small for gestational age (SGA), sex, and Apgar scores at 1 and 5 min. The following clinical information was collected: RDS, BPD, necrotizing enterocolitis (NEC), sepsis, IVH, PVL, retinopathy of prematurity (ROP), duration of invasive ventilation, length of NICU admission, and survival to NICU discharge or death.

### Definitions

PIH was defined as newly diagnosed hypertension in a pregnant woman after 20 weeks of gestation, with systolic blood pressure ≥ 140 mm Hg and/or diastolic blood pressure ≥ 90 mm Hg^2^. The definition of PIH included gestational hypertension, preeclampsia, and eclampsia. Chronic hypertension was defined as a persistent elevation of blood pressure before 20 weeks of gestation or prior to pregnancy. Antenatal corticosteroid administration was defined as the successful completion of a dexamethasone or betamethasone regimen within 7 days before delivery^6^. SGA was defined as the birth weight below the 10th percentile for a GA based on the 2013 Fenton growth charts^19^.

RDS was defined as the presence of acute respiratory insufficiency (grunting, retractions, increased oxygen requirement, tachypnea) with typical radiologic finding after birth and that required surfactant replacement therapy. BPD was diagnosed on the basis of oxygen dependence or respiratory support at 36 weeks of postmenstrual age (PMA) or at NICU discharge, corresponding to moderate to severe BPD using the severity-based definition of BPD in the National Institutes of Health consensus^20^. NEC was defined as stage 2 or higher NEC according to the modified Bell’s staging criteria^21^. Severe IVH was defined as grade III or IV IVH according to the criteria of the Papile classification system, and the worst grading result during hospitalization was recorded^22^. PVL was diagnosed based on the results of brain ultrasound or magnetic resonance imaging findings before NICU discharge. ROP was defined as stage 3 or higher ROP according to the International Committee for the Classification of Retinopathy of Prematurity^23^. Invasive mechanical ventilation included conventional mechanical ventilation and high-frequency oscillatory ventilation.

### Statistical analysis

Continuous variables are expressed as the means ± standard deviations, and categorical variables are expressed as numbers and proportions. Comparisons of continuous variables between the groups were performed using Student’s t test for normally distributed variables and the Mann–Whitney U test for variables with non-normal distributions. Categorical variables were compared using Pearson’s chi-square test. Multivariable logistic regression analysis was performed to adjust for potential confounding variables, and adjusted odds ratios (aORs) and their 95% confidence intervals (CIs) were calculated. The potential confounders adjusted in the multivariable analyses were those identified from the comparison of baseline characteristics. These factors included maternal age, PROM, antenatal steroids, mode of delivery, GA, SGA, and sex. Statistical analyses were performed using SPSS software, version 27.0 (IBM Corp., Armonk NY, USA). A *p* value < 0.05 was considered statistically significant.

### Ethical approval and informed consent

This study was approved by the institutional review board of CHA Bundang Medical Center, CHA University (IRB No. CHAMC 2013-08-082) and the Korean Neonatal Network (2021-058). All methods were performed in accordance with the ethical standards of our institutional research committee and with the 1964 Helsinki declaration and its later amendments.

### Ethics committee/institutional review board

The Korean Neonatal Network registry was approved by the institutional review board and informed consent was obtained from parents of each infant before participation in the KNN registry.

## Results

### Study population

**Figure 1:**
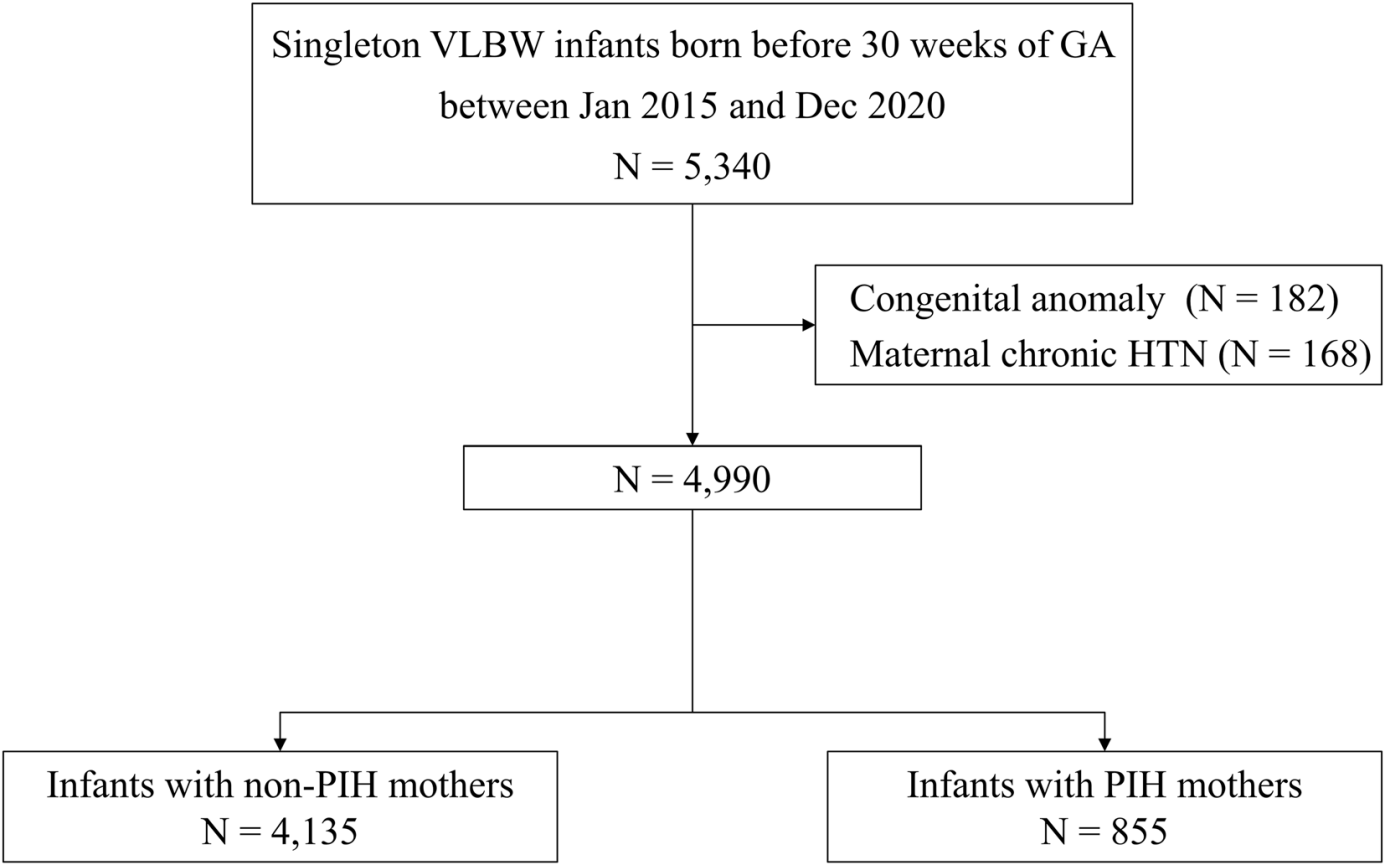
Flow diagram of the study population. *VLBW* Very low birth weight; *GA* Gestational age; *HTN* Hypertension; *PIH* Pregnancy-induced hypertension.

Information on the study population is presented in Fig. 1. Overall, 5340 singleton infants with VLBW born before 30 weeks of GA were registered in the KNN from January 2015 to December 2020. From these infants, we excluded infants with major congenital anomalies (n = 182) and infants born to mothers with chronic hypertension (n = 168). From the remaining 4990 infants, mean GA was 27^+0^ ± 1^+5^ weeks, and mean birth weight was 970 ± 269 g. Of these 4990 infants, 855 (17.1%) infants were born to PIH mothers, and 4135 (82.9%) infants were born to non-PIH mothers.

### Comparison of the baseline characteristics between infants with PIH mothers and infants with non-PIH mothers

The baseline maternal and neonatal characteristics were compared between infants with PIH mothers and infants with non-PIH mothers (Table 1). The mothers with PIH were significantly older and had a higher rate of complete antenatal steroid use than the non-PIH mothers. Maternal PROM occurred more often in non-PIH mothers than PIH mothers. The infants with PIH mothers were born at a higher mean GA and had a lower mean birth weight, a higher rate of SGA, and were less likely to be male than infants with non-PIH mothers. Infants with PIH mothers were more often delivered via cesarean section and had a higher 5-min Apgar score than infants with non-PIH mothers.

**Table 1:** Comparison of the baseline characteristics between infants with PIH mothers and infants with non-PIH mothers.

	Infants with non-PIH mothers N = 4135	Infants with PIH mothers N = 855	*p-*value
Maternal age (years)	33.3 ± 4.6	34.1 ± 4.4	< 0.001
Maternal diabetes (%)	372 (9.0)	95 (11.1)	0.061
Maternal PROM (%)	2149 (52.5)	45 (5.3)	< 0.001
Antenatal corticosteroids (%)	1860 (45.6)	447 (52.7)	< 0.001
Caesarean section (%)	2779 (67.2)	826 (96.6)	< 0.001
Gestational age (weeks^+days^)	27^+0^ ± 1^+6^	27^+5^ ± 1^+4^	< 0.001
Birth weight (g)	996 ± 266	850 ± 249	< 0.001
SGA (%)	200 (4.8)	245 (28.7)	< 0.001
Male (%)	2217 (53.6)	391 (45.7)	< 0.001
1-min Apgar score	4.2 ± 2.0	4.1 ± 1.9	0.276
5-min Apgar score	6.4 ± 1.9	6.6 ± 1.9	0.016

Values are presented as means ± SDs or numbers (%). *PIH* Pregnancy-induced hypertension; *PROM* Premature rupture of membrane; *SGA* Small for gestational age; *SD* Standard deviation.

### Comparison of neonatal mortality and morbidities between infants with PIH and non-PIH mothers

The numbers and rates of neonatal mortality and morbidities between infants with PIH and non-PIH mothers are shown in Table 2. The mortality rate during NICU admission was similar for infants with PIH mothers and infants with non-PIH mothers. Infants with PIH mothers had significantly higher rates of RDS, BPD, and severe BPD and significantly lower rates of severe IVH, PVL, and ROP. There were no differences in the duration of invasive ventilation or length of NICU admission between infants with PIH mothers and infants with non-PIH mothers.

**Table 2:** Comparison of neonatal mortality and morbidities between infants with PIH mothers and non-PIH mothers.

	Infants with non-PIH mothers N = 4135	Infants with PIH mothers N = 855	*p-*value
Respiratory distress syndrome (%)	3875 (93.7)	824 (96.4)	0.002
Bronchopulmonary dysplasia (%)	1514 (44.7)	359 (49.2)	0.027
Moderate bronchopulmonary dysplasia (%)	524 (12.7)	89 (10.4)	0.067
Severe bronchopulmonary dysplasia (%)	990 (23.9)	270 (31.6)	< 0.001
Necrotizing enterocolitis (≥ stage II) (%)	381 (9.3)	84 (9.9)	0.607
Severe intraventricular hemorrhage (%)	482 (12.2)	81 (9.5)	0.002
Periventricular leukomalacia (%)	389 (9.8)	50 (6.0)	< 0.001
Sepsis (%)	1059 (25.8)	204 (23.9)	0.280
Retinopathy of prematurity (≥ stage 3) (%)	625 (18.1)	103 (13.9)	0.006
Death during the NICU admission (%)	971 (23.5)	175 (20.5)	0.061
Duration of invasive mechanical ventilation (days)	20.5 ± 31.3	21.9 ± 33.0	0.230
Length of NICU admission (days)	78.3 ± 48.2	81.1 ± 47.3	0.112

Values are presented as means ± SDs or numbers (%). *PIH* Pregnancy-induced hypertension; *NICU* Neonatal intensive care unit; *SD* Standard deviation.

The multivariable logistic regression analyses evaluating the effect of PIH on mortality and neonatal morbidities are shown in Table 3. After adjusting for potential confounders, infants with PIH mothers had significantly higher odds for RDS (OR 1.983; 95% CI 1.285–3.061, *p* = 0.002), BPD (OR 1.458; 95% CI 1.190–1.785, *p* < 0.001), and severe BPD (OR 1.411; 95% CI 1.163–1.713, *p* < 0.001) than infants with non-PIH mothers. No significant differences in severe IVH, PVL, ROP, or death during NICU admission were observed between infants with PIH mothers and infants with non-PIH mothers in an adjusted comparison.

**Table 3:** Adjusted odds ratios and *p*-values for neonatal mortality and morbidities of infants with PIH mothers compared to those of infants with non-PIH mothers.

	Adjusted OR*(95% CI)	Adjusted *p-*value*
Respiratory distress syndrome	1.983 (1.285–3.061)	0.002
Bronchopulmonary dysplasia	1.458 (1.190–1.785)	< 0.001
Moderate bronchopulmonary dysplasia	0.881 (0.671–1.158)	0.365
Severe bronchopulmonary dysplasia	1.411 (1.163–1.713)	< 0.001
Necrotizing enterocolitis (≥ stage II)	1.254 (0.926–1.699)	0.144
Severe intraventricular hemorrhage	1.043 (0.762–1.427)	0.793
Periventricular leukomalacia	0.770 (0.546–1.086)	0.137
Sepsis	0.989 (0.806–1.213)	0.915
Retinopathy of prematurity (≥ stage 3)	0.891 (0.667–1.189)	0.432
Death during the NICU admission	1.101 (0.867–1.398)	0.431

*The ORs and *p*-values were calculated using logistic regression analysis with adjustment for maternal age, maternal premature rupture of membrane, antenatal steroids, mode of delivery, gestational age, small for gestational age, and sex. *PIH* Pregnancy-induced hypertension; *OR* Odds ratio; *CI* Confidence interval; *NICU* Neonatal intensive care unit.

## Discussion

In this population-based study of singleton VLBW infants born before 30 weeks of gestation, infants with PIH mothers had significantly higher odds of RDS, BPD, and severe BPD than infants with non-PIH mothers after adjusting for potential confounders, while there were no significant differences in severe IVH, PVL, ROP, or death during NICU admission between infants with PIH mothers and infants with non-PIH mothers.

Although the pathophysiology of PIH remains uncertain, inadequate placental implantation and abnormal vascularization may play certain roles in the development of PIH^24^. Inadequate placental implantation leads to uteroplacental ischemia. As a consequence of uteroplacental ischemia, angiogenic or antiangiogenic factors, reactive oxygen species and inflammatory cytokines in the bloodstream of mothers with PIH can cross the placenta, reach the fetus and possibly affect the developing lungs^25,26^.

The association between maternal PIH and RDS in preterm infants remains controversial^10,11,27–29^. A cohort study in the Netherlands reported the protective effect of preeclampsia on RDS in late preterm infants^28^. Another single-center cohort study including preterm infants born at 23–28 gestational weeks demonstrated that preeclampsia increased the risk of RDS^29^. We can infer that different study populations and different onset of maternal PIH could be a reason for these discrepancies among the studies. Similar to several studies examining the association between maternal PIH and RDS in preterm infants, we also showed a twofold increased risk of RDS in infants born to mothers with PIH, after adjustment for confounding factors^10,11,29^. Wang et al. suggested that the antiangiogenic state in PIH contributes to surfactant dysfunction^10^. Considering RDS is secondary to surfactant insufficiency, and the association between PIH and RDS might be biologically plausible.

After adjustment for confounding factors, we found that the increased risk of BPD in infants with PIH mothers is in accordance with previous studies^8,9^. In particular, infants with PIH mothers had significantly higher odds of severe BPD than infants with non-PIH mothers in this study. Because angiogenesis and alveolar development are interactive in the fetal lung, BPD has been recently recognized as a manifestation of vascular disease of the lungs^30^. Impaired pulmonary vascular growth by altered signaling of angiogenic or antiangiogenic factors derived from mothers with PIH may play a role in the pathogenesis of BPD^31^. However, conflicting results on the effect of maternal PIH on BPD in preterm infants have been reported^8,9,32,33^. An international cohort study specifically examining preterm infants born at 24 to 28 weeks of gestation reported that the risk for BPD increased in infants born to mothers with PIH^8^. A meta-analysis demonstrated that PIH was associated with BPD in preterm infants born at < 29 weeks of gestation^9^. By contrast, a meta-analysis of Australian cohorts showed that maternal preeclampsia did not influence the risk of BPD in extremely low-birth-weight infants^32^. Another large population-based study also reported that maternal preeclampsia was associated with a decreased risk of BPD in VLBW infants^33^. In that study, a negative association between maternal preeclampsia and BPD was shown only in a subgroup with a GA greater than 31 weeks. Considering that the study populations in these reports comprised data from birth weight-based registries, not GA-based registries, these discrepancies might be attributable to different study populations. A high number of SGA infants born at a relatively later GA would be preferentially included in birth weight-based registries. To minimize this bias in the KNN cohort, we only included VLBW infants born earlier than 30 weeks of GA in this study.

No difference was found in mortality before NICU discharge between infants born to PIH mothers and infants born to non-PIH mothers. The association of maternal PIH and mortality in preterm infants has not been consistent^7,14–17,34^. Some studies have demonstrated that PIH is associated with an increased or decreased risk of mortality^7,14–17^. Another study has reported no association between PIH and neonatal mortality in preterm infants, similar to our study^34^. Conflicting results can be accounted for in part by differences in statistical approaches, differences in GA and birth weight ranges, and differences in sample sizes.

Several reports have noted a lower incidence of severe brain injuries, such as IVH or PVL, among preterm infants born to mothers with PIH^8,12,13^. They suggested that PIH may serve some adaptive role for the fetus in the face of uteroplacental dysfunction^35^. However, despite the suggested protective mechanisms, no significant differences were observed after adjusting for confounding factors in the rate of severe IVH and PVL between infants born to PIH mothers and infants born to non-PIH mothers in our study. This finding is in accordance with a previous meta-analysis showing that the rate of IVH and PVL in infants with PIH mothers was comparable to that in infants with non-PIH mothers^9^.

In this study, the overall rate of PIH was 17.1%. The maternal PIH rates in our study were comparable to those in previous studies^14,36^. Considering that the worldwide incidence of PIH is increasing with the rising prevalence of predisposing factors, such as increasing maternal age, obesity, assisted reproductive technologies and diabetes, PIH deserves significant attention^5,7^.

There are several strengths to our study. Our data were derived from a large national cohort prospectively collected in Korea. The KNN database is reliable and has been validated with a high degree of precision. In consideration of the bias that may exist in birth weight-based registries, as mentioned above, we excluded infants with a GA of 30 weeks or more from the study population. Additionally, we only included infants born as singleton in this study, which may have influenced the results by plurality^14^. Our study has some limitations. One limitation of the current study lies in the KNN definition of PIH. The KNN definition of PIH does not discriminate between gestational hypertension, preeclampsia, and eclampsia. It is important to know that a subgroup of PIH, such as gestational hypertension, preeclampsia, and eclampsia, may result in different adverse outcomes for infants. Moreover, the KNN database does not include information regarding aspects of the management of maternal PIH, including fetal monitoring and usage of medications such as magnesium sulfate, that may have affected outcomes for the infants^37^.

## Conclusions

In conclusion, the present study demonstrated that preterm infants with PIH mothers had a higher risk for RDS, BPD, and severe BPD than infants with non-PIH mothers among singleton VLBW infants born before 30 weeks of gestation after adjustment for potential confounding factors, while we found no significant differences in severe IVH, PVL, ROP, or death during NICU admission between infants with PIH and non-PIH mothers. Our data showing the increased risk of neonatal respiratory morbidity will improve the care of preterm infants with PIH mothers. Moreover, further research is required to understand the biological mechanisms that shed light on differences between infants born to mothers with and without PIH.

## Data Availability

The datasets generated and analyzed during the current study are not publicly available. This work was supported by the Research Program funded by the Korea Centers for Disease Control and Prevention. There are ethical restrictions on sharing a deidentified data set unless permitted by the CDC of Korea. Data availability was subjected to the Act on Bioethics and Safety [Law No. 1518, article 18 (Provision of Personal Information)]. Contact for sharing the data or access the data can be possible only through the data committee of Korean neonatal network (http://knn.or.kr) and after permitted by the CDC of Korea. Detail contact information was as follows: data access committee; Yun Sil Chang (yschang@skky.edu), ethics committee; So-Young Kim (sykimped@catholic.ac.kr).

## Abbreviations

PIH: Pregnancy-induced hypertension
GA: Gestational age
RDS: Respiratory distress syndrome
BPD: Bronchopulmonary dysplasia
IVH: Intraventricular hemorrhage
PVL: Periventricular leukomalacia
VLBW: Very low birth weight
KNN: Korean neonatal network
NICU: Neonatal intensive care unit
PROM: Premature rupture of membrane
SGA: Small for gestational age
NEC: Necrotizing enterocolitis
ROP: Retinopathy of prematurity
PMA: Postmenstrual age
OR: Odds ratio
CI: Confidence interval
